# Association of Thyroid-Stimulating Hormone (TSH) Levels With the Prognosis of Patients Undergoing Heart Transplantation: A Retrospective Study

**DOI:** 10.3389/fcvm.2021.720922

**Published:** 2021-10-27

**Authors:** Jiajie Wei, Yingsheng Zhou

**Affiliations:** Department of Endocrinology and Metabolism, Beijing Institute of Heart, Lung and Blood Vessel Diseases, Beijing Anzhen Hospital, Capital Medical University, Beijing, China

**Keywords:** heart, transplantation, thyroid hormone, thyroid-stimulating hormone, survival, prognosis

## Abstract

**Purpose:** To investigate the impact of TSH levels using a more stringent cutoff of subclinical hypothyroidism (i.e., TSH > 2.5 mIU/L) on the short-term complications and long-term prognosis in patients who underwent heart transplantation (HTx).

**Methods:** This is a retrospective study of consecutive patients with end-stage heart failure (HF) who underwent HTx. They were divided into three groups: thyroid-stimulating hormone (TSH) ≤ 2.50 mIU/L (L-TSH), 2.50 < TSH ≤ 4.91 mIU/L (M-TSH), and TSH > 4.91 mIU/L (H-TSH). The outcomes are all-cause death and cardiogenic death.

**Results:** There are 63 (70%) males and 27 (30%) females. Nine (10%) patients died within 1 month after surgery, including five cardiogenic deaths. By 1 year, a total of 19 patients total were dead. The survival rate in the M-TSH group was significantly higher than that of the L-TSH group (*P* = 0.017). After adjusted by variables of sex, age, BMI, diabetes history, hypertension history, the multivariable Cox analysis showed that body mass index (HR = 0.804, 95%CI: 0.680–0.951, *P* = 0.011), and L-TSH (HR = 8.757, 95%CI: 1.786–42.948, *P* = 0.007 vs. M-TSH), and H-TSH (HR = 6.427, 95%CI: 1.137–36.327, *P* = 0.035 vs. M-TSH) were independently associated with all-cause death. The multivariable Cox analysis showed that body mass index (HR = 0.703, 95%CI: 0.564–0.878, *P* = 0.002), and L-TSH (HR = 17.717, 95%CI: 1.907–164.607, *P* = 0.011 vs. M-TSH) were independently associated with cardiogenic death.

**Conclusion:** For patients with end-stage HF undergoing HTx, low and high baseline TSH levels are independently associated with 1-year all-cause death and low baseline TSH levels with cardiogenic death.

## Introduction

Heart transplantation (HTx) has evolved as the “gold standard” therapy for patients with end-stage heart failure (HF) with median survival exceeding 10 years. Advancements in the fields of immunosuppression, infection prophylaxis, and surgical techniques have transformed HTx from what was once considered an experimental intervention into a routine treatment. The number of HTx reported by the International Society of Heart and Lung Transplantation registry worldwide is about 3,500–4,000 annually. As the technology actually stands, HTx is the most effective long-term treatment for advanced HF than the available mechanical circulatory support devices (1–4).

The prevalence of subclinical hypothyroidism (SH) is 3–10%. The prevalence of subclinical hyperthyroidism (SHr) is 0.7–9.7%. Thyroid hormones affect cardiac electrophysiology, contractility, and vasculature. SH is associated with an increased risk of coronary heart disease (CHD), especially in subjects under 65 (5–7). An independent association was found between fT4 and adverse outcomes after HTx, and perioperative low fT4 levels could be a prognostic marker of adverse outcomes in HTx (8).

On the other hand, thyroid-stimulating hormone (TSH) is currently the most important indicator for screening thyroid dysfunction because it is the most sensitive thyroid function indicator. There is still a controversy about the TSH threshold that defines “subclinical hypothyroidism”. In patients with overt hypothyroidism, the lack of T4 feedback leads to TSH levels >20 mIU/L, whereas in milder or subclinical hypothyroidism, the TSH levels are 3–20 mIU/L with normal T4 and T3 levels. For example, severe subclinical hypothyroidism has been defined as TSH ≥ 10 mIU/L, while mild subclinical hypothyroidism as TSH < 10 mIU/L (9, 10). The National Academy of Clinical Biochemistry has proposed a cutoff of 2.5 mIU/L to distinguish between euthyroidism and preclinical hypothyroidism. Based on the stringent criteria to discriminate euthyroidism and preclinical hypothyroidism, the authors hypothesized that different TSH levels could affect outcomes of patients diagnosed with HF and who underwent HTx.

Therefore, this study aimed to investigate the impact of TSH levels using a more stringent (or sensitive) cutoff of subclinical hypothyroidism (i.e., TSH > 2.5 mIU/L) on the short-term postoperative complications and long-term prognosis in patients who underwent HTx.

## Materials and Methods

### Study Design and Patients

This retrospective study included consecutive patients diagnosed with end-stage HF and who underwent HTx at Beijing Anzhen Hospital from January 2010 to December 2018. The study was approved by the ethics committee of Beijing Anzhen Hospital. The requirement for individual consent was waived by the committee.

The end-stage heart disease classification was based on the ACC/AHA HF guidelines (11) and the heart function classification of the New York Society of Cardiology (12). This study included patients with HF with clinical symptoms of grade III–IV, which is ineffective in medical treatment and requires special interventional treatment (13). The exclusion criteria were 1) patients taking drugs such as amiodarone, thyroid hormone, or anti-thyroid function drugs in the past month or 2) patients who underwent iodine contrast examination within 1 month.

### Definitions of Thyroid Statuses

In Beijing Anzhen Hospital, the normal reference of TSH range in 0.49–4.91 mIU /L, so cut-off value was set as 4.91 mIU /L. Thyroid statuses were defined as follows: 1) euthyroid state: TSH of 0.49–4.91 mIU/L with FT4 7.8–21.0 pmol/L; 2) subclinical hypothyroidism: TSH > 4.91 mIU/L with FT4 7.8–21.0 pmol/L; 3) subclinical hyperthyroidism: TSH < 0.49 mIU/L with FT4 7.8–21.0 pmol/L; 4) hypothyroidism: TSH > 4.91 mIU/L and FT4 <7.8 pmol/L (14); 5) hyperthyroidism: TSH < 0.49 mIU/L and FT4 >21.0 pmol/L.

In this study, based on the normal range of thyroid function in our hospital and referring to previous literature reports (15, 16), all patients were divided into three groups: TSH ≤ 2.50 mIU/L (L-TSH, lower levels of TSH within the reference range), 2.50 < TSH ≤ 4.91 mIU/L (M-TSH, levels of TSH within the reference range), and TSH > 4.91 mIU/L (H-TSH).

### Follow-Up

The most common complications of the selected patients within 1 month after the operation were recorded: acute infection, infectious pericardial effusion, acute cardiac tamponade, severe arrhythmia, acute rejection, acute renal failure, etc. Debridement, thoracotomy, hemodialysis, pacemaker implantation, extracorporeal membrane oxygenation (ECMO), and death were also recorded. The postoperative follow-up was 1 year, and the survival status, cardiac rehospitalization, and cardiovascular events were recorded.

### Data Collection

The patient preoperative data were collected from the hospital's database, including age, sex, height, weight, body mass index (BMI), systolic blood pressure, diastolic blood pressure, smoking history, drinking history, comorbidities (hypertension, diabetes, and stroke), liver function, kidney function, blood lipids, blood uric acid, blood sugar, and thyroid function. The perioperative data were collected, including operation time, postoperative mechanical ventilation time, intensive care unit (ICU) stay, and total hospital stay after transplantation. An automatic biochemical analyzer (AU5400, Beckman, Brea, CA, USA) was used to measure the biochemistry indicator.

### Outcomes

The outcomes are all-cause death (death caused by any cause) and cardiogenic death (death caused by primary or secondary cardiac causes except for non- cardiogenic causes or other external causes).

### Statistical Analysis

SPSS 22.0 (IBM Corp., Armonk, NY, USA) was used for statistical analysis. The continuous data are presented as means ± standard deviations for those with a normal distribution, or medians and interquartile range (IQR; P25, P75) for those with a non-normal distribution. Categorical data are presented as *n* (%). The comparison among groups was performed by analysis of variance and the LSD *post hoc* test or using the Kruskal-Wallis non-parametric test, as appropriate. The Kaplan-Meier method and the log-rank test were used for univariable survival analysis. The Cox proportional hazards regression model was used to analyze multiple factors. Two-sided *P*-values < 0.05 were considered statistically significant.

## Results

### Characteristics of the Patients

**Figure 1:**
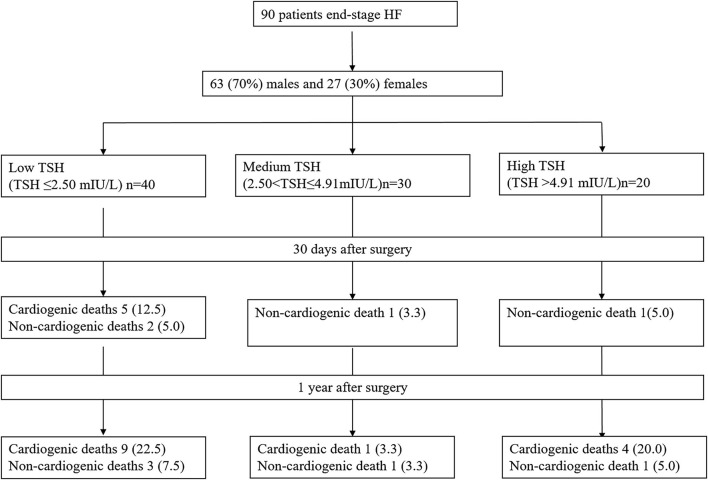
Patient flowchart.

We retrospectively included 90 consecutive patients with end-stage HF who underwent HTx at Beijing Anzhen Hospital from January 2010 to December 2018(Figure 1). Table 1 presents the characteristics of the patients. There are 63 (70%) males and 27 (30%) females. Among the primary heart diseases, there are 60 patients with cardiomyopathy (66.7%, 40 males and 20 females), 18 with coronary atherosclerotic heart disease (20.0%, 16 males and two females), and six with valvular disease (6.7%, three males and three females), four with congenital heart disease (4.4%, three males and one female), and two with malignant cardiac tumor (2.2%, one male and one female). Twenty-five patients (27.8%) have a history of thyroid disease, including one case of hyperthyroidism (1.1%, female, 68 years old), six of hypothyroidism (6.7%, three males and three females), 15 cases of subclinical hypothyroidism (16.7%, 13 males and two females), and three cases of subclinical hyperthyroidism (3.3%, two males and one female). There are no statistically significant differences in sex, age, creatinine, transaminase, blood lipids, blood uric acid, type 2 diabetes, hypertension, and stroke among the three groups. There are significant differences in body weight (*P* = 0.034), BMI (*P* = 0.023), and fasting blood glucose (FBG) (*P* = 0.045) among the three groups (Table 1).

**Table 1:** Characteristics of heart transplantation patients according to TSH levels.

**Clinical findings**	**TSH Groups**			***P***
	**Low TSH** **(TSH ≤2.50 mIU/L)** ***n*** **= 40**	**Medium TSH** **(2.50 < TSH ≤ 4.91 mIU/L)** ***n*** **= 30**	**High TSH** **(>4.91 mIU/L)** ***n*** **= 20**	
Age (years)	49.8 ± 12.8	44.7 ± 13.5	45.0 ± 15.8	0.163
Sex				0.452
Male, *n* (%)	28 (70)	19 (63)	16 (80)	
Weight (kg)	67.9 ± 13.5^b^	61.1 ± 14.2	70.4 ± 11.1^b^	0.034
BMI (kg/m^2^)	23.8 ± 3.8^b^	21.7 ± 3.9	24.1 ± 2.7^b^	0.023
NYHA, n (%)				0.827
III	6 (15)	6 (20)	4 (20)	
IV	34 (85)	24(80)	16 (80)	
History of thyroid disease, *n* (%)				<0.001
Hyperthyroidism	1 (2.5)	0	0	0.532
Hypothyroidism	2 (5.0)	1 (3.3)	3 (15)	0.229
Subclinical hyperthyroidism	3 (7.5)	0	0	0.144
Subclinical hypothyroidism	0	0	15 (75)^aa^, ^bb^	<0.001
No history of thyroid disease	34 (85)	29 (97)	2 (10)^aa^, ^bb^	<0.001
History of heart disease, n (%)				0.277
Valvular disease	3 (7.5)	1 (3.3)	2 (10)	0.626
Coronary heart disease	12 (30)	3 (10)	3 (15)	0.096
Congenital heart disease	3 (7.5)	1 (3.3)	0	0.387
Dilated cardiomyopathy	22 (55)	24 (80)	14 (70)	0.084
Other	0	1 (3.3)	1 (5)	0.409
Smoking history, *n* (%)	13 (33)	12 (40)	7 (35)	0.809
Alcohol history, *n* (%)	11 (28)	10 (33)	6 (30)	0.870
Diabetes history, *n* (%)	9 (23)	2 (6.7)	5 (25)	0.145
Stroke history, *n* (%)	2 (5.0)	2 (6.7)	1 (5.0)	0.948
Hypertension, *n* (%)	12 (30)	5 (17)	6 (30)	0.393
SBP (mmHg)	115 ± 16	111 ± 16	121 ± 17	0.161
DBP (mmHg)	77 ± 12	73 ± 15	77 ± 13	0.585
TSH (mlU/L)	1.41 ± 0.63	3.14 ± 0.57^aa^	6.61 ± 1.41^aa^, ^bb^	<0.001
FT3 (pmol/L)	4.31 ± 0.89	4.47 ± 0.80	4.38 ± 0.69	0.711
FT4 (pmol/L)	14.10 ± 2.76	15.08 ± 3.35	14.48 ± 3.46	0.435
BUN (mmol/L)	8.44 ± 3.04	7.73 ± 2.53	8.62 ± 2.86	0.474
Cr (μmol/L)	89.84 ± 25.49	84.01 ± 23.85	95.09 ± 34.20	0.362
ALT (IU/L)	24.00 (17.00,39.50)	23.50 (15.50,39.00)	24.50 (12.00,50.50)	0.893
AST (IU/L)	26.00 (18.00,33.75)	24.50 (21.00,35.00)	27.50 (22.50,31.00)	0.769
UA (μmol/L)	485.48 ± 181.88	484.61 ± 176.89	473.10 ± 123.36	0.871
TG (Immol/L)	1.17 (0.80,1.53)	0.98 (0.77,1.66)	1.02 (0.76,1.34)	0.687
TC (mmol/L)	4.27 ± 1.24	4.28 ± 0.88	4.58 ± 1.49	0.771
HDL-C (mmol/L)	0.98 ± 0.28	1.00 ± 0.27	1.08 ± 0.39	0.430
LDL-C (mmol/L)	2.72 ± 0.97	2.75 ± 0.71	2.80 ± 0.86	0.938
FPG (mmol/L)	5.57 (5.02,6.84)	5.10 (4.41,6.28)	6.39 (5.18,7.40)^b^	0.045
Mechanical ventilation time (h)	20.00 (16.00,24.00)	22.50 (13.75,39.25)	41.00 (23.00,66.25)^aa^	0.009
Intensive care time (h)	40.50 (26.00,72.00)	49.00 (29.50,87.00)	80.50 (49.25,90.50)^a^	0.022
Hospital stay after transplantation (d)	28.00 (20.25,39.50)	28.50 (23.75,36.50)	23.00 (21.00,35.25)	0.497

*Continuous data are presented as means ± standard deviations or interquartile ranges*.

a *P < 0.05*,

aa *P < 0.01 vs. the TSH ≤ 2.50 mlU/L group*.

b *P < 0.05*,

bb *P < 0.01 vs. the 2.50 < TSH ≤ 4.91 mlU/L group*.

*BMI, body mass index; NYHA, New York Heart Association; TSH, thyroid-stimulating hormone; FT3, freed triiodothyronine; FT4, free thyroxine; BUN, blood urea nitrogen; SBP, systolic blood pressure; DBP, diastolic blood pressure; Cr, creatinine; ALT, alanine transaminase; AST, aspartate transaminase; UA, uric acid; TG, triglycerides; TC, total cholesterol; HDL-C, high-density lipoprotein cholesterol; LDL-C, low-density lipoprotein cholesterol; FPG, fasting plasma glucose*.

### Perioperative Recovery

There are no significant differences in the length of hospitalization days after transplantation among the TSH groups, but the length of mechanical ventilation (*P* = 0.009) and the intensive care unit stay (*P* = 0.022) are significantly different among the TSH groups, with those durations being longer in the TSH > 4.91 mIU/L (Table 1).

### Short-Term (Within 1 Month After Surgery) Prognosis

Table 2 presents the short-term outcomes. The 90 patients were followed for 1 month after the operation. Nine (10%) patients died, including five cardiogenic deaths, one from liver and kidney failure, two from gastrointestinal bleeding, and one from toxic shock. Four patients underwent repeat thoracotomy, of which one is due to chylothorax, and three were due to bleeding. Debridement was performed in two patients with poor wound healing. There are six cases of continuous renal replacement therapy (CRRT) after the operation. There are three cases of ECMO.

**Table 2:** Adverse events during follow-up.

**Follow-up adverse reactions and treatment**	**TSH groups**			***P***
	**Low TSH** **(TSH ≤2.50 mIU/L)** ***n*** **= 40, (%)**	**Medium TSH** **(2.50 < TSH ≤ 4.91 mIU/L)** ***n*** **= 30, (%)**	**High TSH** **(>4.91 mIU/L)** ***n*** **= 20, (%)**	
**30 days after surgery**				
Acute infection	2 (5.0)	0	0	0.278
Infectious pericardial effusion	0	0	0	
Acute cardiac tamponade	2 (5.0)	0	1 (5.0)	0.460
Severe arrhythmia	0	0	0	
Acute rejection	0	0	0	
Acute renal failure	6 (15.0)	0	0	0.018
Debridement	1 (2.5)	0	1 (5.0)	0.495
re-thoracotomy	3 (7.5)	0	1 (5.0)	0.318
Continuous renal replacement therapy	6 (15.0)	0	0	0.018
Pacemaker implantation	0	0	0	
Use of ECMO	3 (3)	0	0	0.144
Cardiogenic death*	5 (12.5)	0	0	0.037
Non-cardiogenic death^#^	2 (5.0)	1 (3.3)	1 (5.0)	0.937
**1 year after surgery**				
Cardiovascular events	0	0	0	
Heart failure re-hospitalization	1 (2.5)	1 (3.3)	2 (10.0)	0.387
Coronary intervention or surgery	1 (2.5)	1 (3.3)	0	0.726
Cardiogenic death*	9 (22.5)	1 (3.3)	4 (20.0)	0.075
Non-cardiogenic death#	3 (7.5)	1 (3.3)	1 (5.0)	0.747

* *Cardiogenic death: including graft failure, heart failure, and graft rejection*.

# *Non-cardiogenic death: including septic shock, renal failure, upper gastrointestinal hemorrhage, and multiple organ failure*.

### Long-Term (12 Months After Surgery) Prognosis

All patients were followed for 12 months after surgery. Nineteen (21.1%) patients died in total, including 10 new deaths that occurred after 1 month after surgery, including two from renal or multiple organ failure, four from rejection, one from a lung infection, and three from other reasons. Regarding non-fatal complications, there are six patients with cardiac rejection, eight with renal insufficiency, six with a lung infection (including two with *Pneumocystis carinii*), five with diabetes, one with stroke, and two others.

### Univariable and Multivariable Analyses

**Figure 2:**
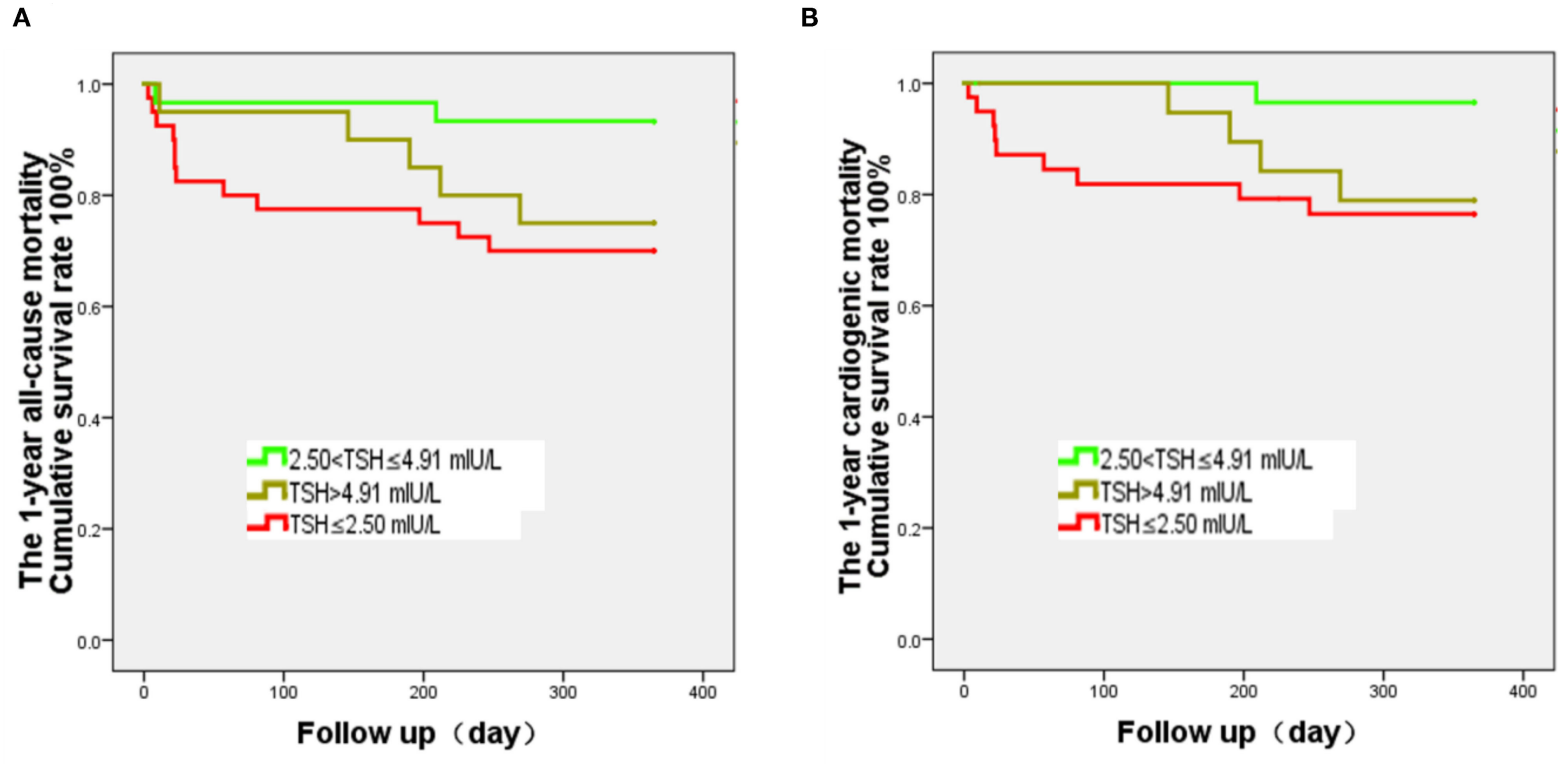
Kaplan-Meier analysis of all-cause and cardiogenic mortality after heart transplantation and according to the thyroid-stimulating hormone (TSH) levels. The curves were analyzed using the log-rank test. The follow-up was 1 year. **(A)** The 1-year all-cause mortality of the 2.50 < TSH ≤ 4.91 mIU/L group is significantly lower than that of the TSH ≤ 2.50 mIU/L group (*P* = 0.017), while there is no difference of survival rate between the 2.50 < TSH ≤ 4.91 mIU/L and TSH > 4.91 mIU/L groups (*P* = 0.074). **(B)** The 1-year cardiogenic mortality of the 2.50 < TSH ≤ 4.91 mIU/L group is significantly lower than that of the TSH ≤ 2.50 mIU/L group (*P* = 0.022), while there is no difference in mortality rate between the 2.50 < TSH ≤ 4.91 mIU/L and TSH > 4.91 mIU/L groups (*P* = 0.052).

The results of the survival analyses showed that the survival curves of the three TSH groups are significantly separated at 1 year of follow-up (Figures 2A,B). The survival rate in the M-TSH group is significantly higher than that of the L-TSH group (*P* = 0.017).

For the TSH categories in the multivariable analyses, the M-TSH group was the reference group. The multivariable Cox analysis showed that body mass index (HR = 0.804, 95%CI: 0.680–0.951, *P* = 0.011), and L-TSH (HR = 8.757, 95%CI: 1.786–42.948, *P* = 0.007 vs. M-TSH), and H-TSH (HR = 6.427, 95%CI: 1.137–36.327, *P* = 0.035 vs. M-TSH) were independently associated with all-cause death (Table 3). The multivariable Cox analysis showed that body mass index (HR = 0.703, 95%CI: 0.564–0.878, *P* = 0.002) and L-TSH (HR = 17.717, 95%CI: 1.907–164.607, *P* = 0.011 vs. M-TSH) were independently associated with cardiogenic death (Table 3).

**Table 3:** Univariable and multivariable Cox regression analyses during the 1-year follow-up.

**Variables**	**HR**	**95%CI**	**P**	**HR**	**95%CI**	**P**
	**Univariable**			**Multivariable**		
All-cause death*						
Sex	1.268	0.457–3.520	0.649	1.942	0.617–6.110	0.256
Age	1.026	0.990–1.063	0.156	1.028	0.992–1.066	0.129
BMI	0.933	0.825–1.054	0.264	0.804	0.680–0.951	0.011
History of thyroid disease (yes vs. no)	1.228	0.907–1.664	0.184			
TSH category						
2.50 < TSH ≤ 4.91 mIU/L	Reference	–	–	Reference	–	–
TSH ≤ 2.50 mIU/L	5.199	1.163–23.242	0.031	8.757	1.786–42.948	0.007
TSH > 4.91 mIU/L	3.949	0.766–20.357	0.101	6.427	1.137–36.327	0.035
Diabetes history (yes vs. no)	1.314	0.445–4.042	0.602	1.191	0.351–4.037	0.779
Hypertension (yes vs. no)	1.020	0.367–2.832	0.970	0.858	0.295–2.495	0.778
**Cardiogenic death** #						
Sex	0.815	0.273–2.434	0.715	1.306	0.352–4.837	0.690
Age	1.016	0.976–1.058	0.428	1.026	0.984–1.069	0.231
BMI	0.873	0.756–1.009	0.066	0.703	0.564–0.878	0.002
History of thyroid disease (yes vs. no)	1.344	0.954–1.892	0.091	1.430	0.867–2.359	0.161
TSH category						
2.50 < TSH ≤ 4.91 mIU/L	Reference	–	–	Reference	–	–
TSH ≤ 2.50 mIU/L	7.966	1.009–62.914	0.049	17.717	1.907–164.607	0.011
TSH > 4.91 mIU/L	6.367	0.711–56.976	0.098	7.212	0.488–106.512	0.150
Diabetes history (yes vs. no)	1.389	0.387–4.980	0.614	2.441	0.516–11.549	0.260
Hypertension (yes vs. no)	1.151	0.361–3.672	0.812	0.736	0.168–3.217	0.683

*HR, hazard ratio; CI, confidence interval; TSH, thyroid-stimulating hormone*.

* *: multivariable analysis was adjusted by variables of sex, age, BMI, Diabetes history (yes vs. no), Hypertension history (yes vs. no)*.

# *: multivariable analysis was adjusted by variables of sex, age, BMI,History of thyroid disease (yes vs. no), Diabetes history (yes vs. no), Hypertension history (yes vs. no)*.

## Discussion

Low thyroid hormone levels are associated with a poor prognosis of HF (17–22), but rare studies examined thyroid hormones on prognosis after HTx. Therefore, this study aims to investigate the impact of TSH levels using a more stringent cutoff of subclinical hypothyroidism (i.e., TSH > 2.5 mIU/L) on the short-term complications and long-term prognosis in patients who underwent HTx. The results strongly suggest that for patients with end-stage HF undergoing HTx, low and high baseline TSH levels were independently associated with 1-year all-cause death, and low baseline TSH levels were independently associated with cardiogenic death.

There are still some different controversies about the normal range of TSH. An analysis of the Nord-Trondelag Health Study (HUNT Study), one of the largest longitudinal health studies in the world with extensive phenotypic data linked to regional and national disease registers, identified that higher TSH levels within the reference range were associated with higher mortality from coronary heart disease in females (23). These results suggest that the entire spectrum of hypothyroidism, from high-normal serum TSH to mild and frankly elevated serum TSH, is associated with relevant metabolic risk factors, coronary heart disease events, and mortality. Moreover, L-thyroxine has been found to exert a beneficial effect on atherogenic lipid profile and impaired vascular function in patients with TSH levels between 2.5 and 4.5 mIU/L (9, 17). A study showed a significant protective association of subclinical hypothyroidism with better outcomes and lower mortality after cerebral ischemic stroke; possible explanations for this association are ischemic preconditioning, reduced adrenergic tone, and hypometabolic state (18). The normal range of TSH in our hospital is 0.49–4.91mIU/L. Patients with serum TSH levels >2.50 mU/l are more likely to be detected with thyroid autoantibodies, and it has been suggested that TSH > 2.50 mIU/L can be defined as subclinical hypothyroidism (10), but TSH cutoff of hypothyroidism at TSH > 4.50 mIU/L has been suggested (19). There is no report on the influence of different levels of normal TSH range on patients' prognosis in the perioperative period of HTx.

This study found that 70% of end-stage heart disease patients undergoing HTx are men and that most patients had cardiomyopathy (66.7%). In this study, non-ischemic cardiomyopathy, such as dilated cardiomyopathy, is the main cause of HF. There were 33.3% of patients diagnosed with thyroid disease, and 16.7% of patients had subclinical hypothyroidism. The Rotterdam Study showed that subclinical hypothyroidism, like hyperglycemia, hypertension, and hyperlipidemia, is an independent risk factor for ischemic heart disease (20). Conversely, it can be inferred that the proportion of patients with hypothyroidism in patients with HF due to poor prognosis of heart disease may be higher than the general population. It may be the reason for the higher morbidity of patients with thyroid disease in this study. In recent years, other studies have confirmed that the proportion of subclinical hypothyroidism in patients with chronic HF is significantly higher than that of the normal population (20).

The differences in weight, BMI, and FBG might be related to the fact that patients with subclinical hypothyroidism are more likely to have multiple metabolic disorders and weight gain (21, 22, 24). It may also be related to the small sample size of this study. In the future, more the sample size will be expanded to observe further the differences in the basic conditions of the three groups.

During the perioperative recovery, ICU stay and mechanical ventilation duration were significantly lower in the TSH ≤ 2.5 mIU/L group than in the TSH > 4.91 mIU/L group, indicating a worse perioperative recovery in patients with hypothyroidism. Therefore, according to this study's results, alleviating subclinical hypothyroidism during the perioperative period might shorten the time of mechanical ventilation and the time of intensive care in patients, which is conducive to recovery.

The 90 patients were followed for 1 month after surgery. Nine patients died, of which seven patients died from graft failure or other organ failure. At 1 year, there were 19 deaths, including 10 new deaths and 4 deaths due to transplantation heart rejection. With advances in immunosuppressive agents, heart preservation technology, surgical technology, donor and recipient selection, and rejection monitoring, the survival rates of HTx recipients at 30 days and 1 year after surgery are 90 and 86%, respectively (25). In the 1-year follow-up, there were six cardiac rejection cases, eight of renal insufficiency, six of lung infection, and five of diabetes. These complications are considered to be related to the postoperative use of immunosuppressive drugs.

The Kaplan-Meier survival curve analysis found that the 1-year survival rate of the M-TSH group was significantly higher than that of the L-TSH group. The multivariable models for all-cause and cardiogenic death showed that TSH levels were independently associated with death. Chen et al. (26) found that both hyperthyroidism and hypothyroidism increased the mortality of patients with HF, supporting the present study. Li et al. (27) retrospectively analyzed 963 patients with dilated cardiomyopathy in China. They found that subclinical thyroid dysfunction increased patients' mortality and that subclinical hyperthyroidism was an independent risk factor for all-cause death, supporting the present study. This study found no significant difference in the incidence of cardiogenic deaths between the H-TSH and L-TSH groups at 1-year. On the one hand, subclinical hypothyroidism and hypothyroidism with high TSH and lower basal metabolism fail to meet the requirements of maintaining heart function, which might not improve the prognosis. On the other hand, subclinical hyperthyroidism or hyperthyroidism also increases the heart's burden through excessive consumption of basal metabolism. Nevertheless, high serum levels of TSH have a negative correlation with the incidence of cardiovascular death in elderly patients (>85 years old) (28). Therefore, as age increased, maintaining the balance of cardiac function and cardiac load may be an important mechanism that affects the patient's cardiac prognosis.

This study has limitations. The study sample is from a single center and is small, but it represents a relatively large group of patients with HTx in China. In addition, the proportions of patients with subclinical hyperthyroidism, overt hypothyroidism, and overt hyperthyroidism were small. Third, its retrospective design limited the data to those available in the charts. In future studies, increasing the sample size or increasing the numbers of patients with subclinical thyroid diseases (such as hypothyroidism or hyperthyroidism) could provide firmer conclusions. Additional factors associated with thyroid function but that are not routinely measured could be explored.

Patients with low and high baseline TSH levels have higher 1-year mortality, and patients with low baseline TSH levels have higher cardiogenic mortality after HTx than patients with TSH between 2.50 and 4.91 mIU/L. For patients with end-stage HF undergoing HTx, it is necessary to monitor the patient's thyroid function before surgery. HTx patients with TSH levels of 2.50–4.91 mIU/L have a good prognosis and higher long-term survival, providing some evidence for preoperative and postoperative management of patients with end-stage HF planned for HTx. Still, the effect of TSH on the complications and mortality of HTx patients with a diagnosis of thyroid disease needs further study.

## Data Availability Statement

The raw data supporting the conclusions of this article will be made available by the authors, without undue reservation.

## Ethics Statement

The studies involving human participants were reviewed and approved by the Ethics Committee of Beijing Anzhen Hospital. Written informed consent for participation was not required for this study in accordance with the national legislation and the institutional requirements.

## Author Contributions

JW performed this study design, data collection, data analysis, and paper draft. YZ was the primary investigator of the project and revised the paper. All authors reviewed the results and approved the final version of the manuscript.

## Funding

This study was funded by the National Natural Science Foundation of China (81641027) and the Beijing Science and Technology Project (Z131100004013044).

## Conflict of Interest

The authors declare that the research was conducted in the absence of any commercial or financial relationships that could be construed as a potential conflict of interest.

## Publisher's Note

All claims expressed in this article are solely those of the authors and do not necessarily represent those of their affiliated organizations, or those of the publisher, the editors and the reviewers. Any product that may be evaluated in this article, or claim that may be made by its manufacturer, is not guaranteed or endorsed by the publisher.
